# P-787. Potential Impact of Repeating Unexpected Positive Interferon Gamma Release Assays

**DOI:** 10.1093/ofid/ofae631.981

**Published:** 2025-01-29

**Authors:** Michael Kiritsy, Nicholas A Turner, Sofia Zavala, Jason E Stout

**Affiliations:** Duke University, Durham, North Carolina; Duke University Medical Center, Durham, North Carolina; Duke University Medical Center, Durham, North Carolina; Duke University School of Medicine, Durham, North Carolina

## Abstract

**Background:**

Interferon gamma release assays (IGRAs) are more specific for tuberculosis infection than the tuberculin skin test but also have higher test-retest variability. False positive tests may occur in low prevalence populations. As such, repeating unexpected positive tests (positive tests in patients without exposure risk) is recommended.

**Table:**
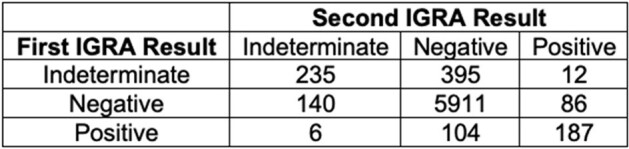
Initial and repeat IGRA results

**Methods:**

We performed a retrospective electronic record review of all patients for whom a QuantiFERON (IGRA) was performed in Duke University Health System between 10/1/2014-5/3/2024. We examined patterns of repeat testing and ascertained the potential clinical impact of repeat testing.

**Results:**

In total, 59,485 IGRAs were performed on 49,850 patients during the study period. 7,076 patients had repeat IGRA testing (median 2 IGRAs, range 2-9). Of these, 6137 had negative, 297 positive, and 642 indeterminate results on the first test. When repeat testing was performed, 743 (11%) patients had a different result on the second test (Table). Of patients with initial positive results, 104/297 (35%) had a negative result on the second test, which was performed a median of 110 days later (IQR 16-420 days). 86 patients had a negative first test but a positive second test; of these, 38 had a third test performed that was negative in 27 (71%) and positive in 11 (29%).

**Conclusion:**

In a low-prevalence setting, repeating positive IGRA tests potentially avoided unnecessary preventive treatment in over a third of patients with an initial positive test and in two-thirds of patients with a prior negative test.

**Disclosures:**

**Nicholas A. Turner, MD, MHSc**, PDI: Research contract|Purio Labs: Research contract

